# The Association of Oral Health Status, demographic characteristics and socioeconomic determinants with Oral health-related quality of life among children: a systematic review and Meta-analysis

**DOI:** 10.1186/s12887-020-02371-8

**Published:** 2020-10-22

**Authors:** Ladan Fattah Moghaddam, Mario Vianna Vettore, Azadeh Bayani, Amir-Hossien Bayat, Elahe Ahounbar, Morteza Hemmat, Bahram Armoon, Yadolah Fakhri

**Affiliations:** 1 Department of nursing, faculty of nursing and midwifery, Tehran medical sciences, Islamic Azad University, Tehran, Iran; 2 Department of Social and Preventive Dentistry, Dental School, Federal University of Minas Gerais, Belo Horizonte, Brazil; 3 Student Research Committee, School of Allied Medical Sciences, Shahid Beheshti University of Medical Sciences, Tehran, Iran; 4 Social Determinants of Health Research Center, Saveh University of Medical Sciences, Saveh, Iran; 5 Substance Abuse and Dependence Research Center, University of Social Welfare and Rehabilitation Sciences, Tehran, Iran; 6 Department of Environmental Health Engineering, Hormozgan University of Medical Sciences, Bandar Abbas, Iran

**Keywords:** Oral health, Quality of life, Children

## Abstract

**Background:**

Health-related quality of life (HQoL) indicators are considered valid measures of patient assessment in physical, mental and oral healthcare. This study aimed to examine the evidence on the relationship of oral health status, demographic and socioeconomic characteristics with oral health-related quality of life (OHRQoL) in children.

**Methods:**

Studies in English published up to December 2019 were searched on PsycINFO, PubMed, SciELO, Scopus, and Web of Science databases. Epidemiological studies simultaneously assessing sociodemographic factors related to oral health (age, income, gender, maternal education), oral health measures (orthodontic treatment needs, dental caries and periodontal disease) and OHRQoL in children aged 3–12 years were included. Methodological quality was assessed using a Critical Appraisal Checklist. Meta-analysis was used to estimate pooled measures between sociodemographic factors and oral health measures with OHRQoL.

**Results:**

Eleven articles were included. Lower children’s age (3–5 years vs > 5), gender (girls vs boys), lower income (< 70$ vs ≥ $70), low maternal education (≤ 6 vs > 6 years) were associated with poor OHRQoL among children. Orthodontic treatment needs, dental caries and periodontal diseases were also associated with poor children’s OHRQoL. Meta-regression showed that Human Development Index, sample size, year of publication and participant’s age were relevant aspects that influenced the above mentioned relationships.

**Conclusions:**

Our findings suggest that oral health promotion strategies to improve children’s OHRQoL should consider the social and environmental where they live as well their oral health status. Further longitudinal studies are needed to explore the determinants of OHQoL in children.

## Background

The use of oral health-related quality of life (OHRQoL) in healthcare along with clinical assessment reflects a shift from normative approach to a patient-centred care perspective in the evaluation of oral health and the efficacy of dental treatment [1, 2]. OHRoL indicators assess different dimensions of oral health status of the impact of oral conditions on physical, emotional, social and psychological aspects on a person’s subjective well-being [1, 3]. Previous research showed that OHRQoL may be influenced by oral health conditions, demographic and socioeconomic characteristics, and contextual factors such as political and cultural aspects [4]. Furthermore, children’s oral health status can affect their well-being and quality of life as well as that of their parents.

Dental caries and periodontal disease are relevant public health problems that account for a considerable proportion of the global burden of oral diseases [5]. The etiology of oral diseases are multifactorial and their distribution and severity of oral diseases in children may vary according to family’s socioeconomic condition [6]. Fisher-Owens and colleagues developed a theoretical framework involving relevant predictors of children’s oral health that were grouped into community- family- and child-level influences [7]. They have shown the importance of socio-behavioral and environmental factors on children’s oral health. Of them, family environment plays a central role in children’s oral health [8].

Environmental and sociodemographic factors can influence OHRQoL in children and other age groups [8, 9]. Age [10], sex [11], socioeconomic status [12–14], socio-cultural factors [10, 15, 16], psychosocial factors [17–19] were associated with OHRQoL. For instance, income and family structure were significant predictors of children’s OHRQoL, independently of oral diseases [20]. Several studies suggest that oral diseases can impact on children and adolescents OHRQoL [21–24]. Locker (2007) showed that children from low-income families and those with only one adult in the household were more likely to poor OHRQoL [25]. Children from low-income families also have poor general and oral health than those from better off families [26]. This study aimed to analyze the evidence on the association of oral health conditions (orthodontic treatment needs, dental caries and periodontal disease), demographic and socioeconomic characteristics (age, gender, income, maternal education) with oral health-quality of life in children.

## Methods

The present systematic review was performed in accordance with Preferred Reporting Items for Systematic Reviews and Meta-Analyses (PRISMA) [27, 28].

### Eligibility criteria and PECO terms

Epidemiologic studies concomitantly assessing sociodemographic factors related to oral health, oral health measures and children’s OHRQoL in children were included. The PECO tool using the following terms were used: (a) Participants: children aged 3–12 years; (b) Exposures: orthodontic treatment needs, dental caries and periodontal disease, children’s age, gender, family income, maternal education; (c) Comparison: non-children groups; (d) Outcomes: oral health-related quality of life. Only studies reporting quantitative measures and/or dental indices to assess oral health status (e.g. number of decayed, missing, and filled teeth [DMFT] for dental caries) [29], studies assessing OHRQoL through validated questionnaire, those reporting measures of association and published in English were included. In addition, only studies reporting data on severe oral health conditions, such as malocclusion, dental caries and periodontal disease, were considered. Investigations regarding temporomandibular disorders, erosion, or xerostomia were excluded. Studies comparing OHRQoL measures between genders, those considering the family income threshold of 70 $ and maternal education of 6 years of education were included. Only articles reporting coefficients and odds ratios were included. The methodological quality of the studies was also considered as inclusion criteria. Only studies considered of medium or high quality (at least three points on the Critical Appraisal Checklist for observational studies) were included [30].

### Search strategy and selection of studies

The search strategy was based on MeSH terms according to PECO terms. Papers published up to December 2019 on PsycINFO, PubMed, SciELO, Scopus, Web of Science, and Cochrane electronic databases were screened for inclusion. Table 1 presents the search strategy, including the combination of key words used in the different electronic libraries. Exclusion of duplicate papers was conducted using EndNote X7 software (Thomson Reuters, New York, NY, USA).

**Table 1:** search strategy

Database	Key words
PsycINFO	**(“social Health” OR “physical Health OR Mental Health OR” Quality of Life”) AND (“social determinants of health” OR “Socioeconomic” AND (“Dental Caries” OR “Periodontal Diseases” OR “Periodontitis” OR “DMF Index” OR “Tooth Loss” OR “Edentulism” OR “Dental Status” OR “Oral Health”) AND (“Pediatrics” OR “Child” OR “children” OR “school children” OR “preschool children”)**
Scielo	**social Health [Title words] or physical Health [Title words] or Mental Health [Title words] or Quality of Life [Title words] and social determinants of health [Title words] or Socioeconomic [Title words] and Oral health [Title words] or Dental Caries [Title words] or periodontal [Title words] or DMF Index [Title words] or oral hygiene [Title words] or decayed, missed and filled teeth [Title words] or tooth Loss [Title words] or “Edentulism” [Title words] or “Dental Status” [Title words] or “Oral Health” [Title words] and Pediatrics [Title words] or Child [Title words] or children [Title words] or school children [Title words] or preschool children [Title words]**
Pubmed	#33 Search (((((((social Health [Title]) OR physical Health [Title]) OR Mental Health [MeSH Terms]) OR Quality of Life [MeSH Terms])) AND ((Socioeconomic Factors [MeSH Terms]) OR Social Determinants of Health [MeSH Terms])) AND ((((school children [Title]) OR Child, Preschool [MeSH Terms]) OR Pediatrics [MeSH Terms]) OR child [MeSH Terms])) AND (((((((((Dental Status [Title]) OR edentulism [Title]) OR Oral Hygiene Index [MeSH Terms]) OR Oral Health [MeSH Terms]) OR Tooth Loss [MeSH Terms]) OR DMF Index [MeSH Terms]) OR Periodontitis [MeSH Terms]) OR Periodontal Diseases [MeSH Terms]) OR Dental Caries [MeSH Terms]) #32 Search (Socioeconomic Factors [MeSH Terms]) OR Social Determinants of Health [MeSH Terms] #31 Search Socioeconomic Factors [MeSH Terms] #28 Search Social Determinants of Health [MeSH Terms] #26 Search ((((((((Dental Status [Title]) OR edentulism [Title]) OR Oral Hygiene Index [MeSH Terms]) OR Oral Health [MeSH Terms]) OR Tooth Loss [MeSH Terms]) OR DMF Index [MeSH Terms]) OR Periodontitis [MeSH Terms]) OR Periodontal Diseases [MeSH Terms]) OR Dental Caries [MeSH Terms] #25 Search Dental Status [Title] #24 Search edentulism [Title] #23 Search Oral Hygiene Index [MeSH Terms] #22 Search Oral Health [MeSH Terms] #21 Search Tooth Loss [MeSH Terms] #20 Search DMF Index [MeSH Terms] #19 Search Periodontitis [MeSH Terms] #18 Search Periodontal Diseases [MeSH Terms] #17 Search Dental Caries [MeSH Terms] #16 Search (((school children [Title]) OR Child, Preschool [MeSH Terms]) OR Pediatrics [MeSH Terms]) OR child [MeSH Terms] #15 Search school children [Title] #14 Search Child, Preschool [MeSH Terms] #11 Search Pediatrics [MeSH Terms] #7 Search child [MeSH Terms] #5 Search (((social Health [Title]) OR physical Health [Title]) OR Mental Health [MeSH Terms]) OR Quality of Life [MeSH Terms] #4 Search social Health [Title] #3 Search physical Health [Title] #2 Search Mental Health [MeSH Terms] #1 Search Quality of Life [MeSH Terms]
Scopus	**(TITLE-ABS-KEY (quality AND of AND life**) **OR TITLE-ABS-KEY (mental AND health) OR TITLE-ABS-KEY (physical AND health) OR TITLE-ABS-KEY (social AND health)) AND (TITLE-ABS-KEY (pregnant AND woman) OR TITLE-ABS-KEY (pregnancy) OR TITLE-ABS-KEY (mothers)) AND (TITLE-ABS-KEY (dental AND caries) OR TITLE-ABS-KEY (periodontal AND diseases) OR TITLE-ABS-KEY (periodontitis) OR TITLE-ABS-KEY (dmf AND index) OR TITLE-ABS-KEY (tooth AND loss) OR TITLE-ABS-KEY (oral AND health) OR TITLE-ABS-KEY (oral AND hygiene AND index) OR TITLE-ABS-KEY (edentulism) OR TITLE-ABS-KEY (dental AND status))**
Web of Knowledge	TS = (Quality of Life OR Health related Quality of Life OR Physical health OR mental health OR social health) AND TS = (Dental Caries OR Periodontal Diseases OR Periodontitis OR DMF Index OR Tooth Loss OR Edentulism OR Dental Status OR Oral Health OR Oral Hygiene Index) AND TS = (school children OR Preschool OR Pediatrics OR child)
Cochrane	#1 MeSH descriptor: [Quality of Life] explode all trees #2 MeSH descriptor: [Mental Health] explode all trees #3 (physical Health): ti,ab,kw #3 (social Health): ti,ab,kw #4 #1 OR #2 OR #3 #6 Search school children [Title] #7 Search Child, Preschool [MeSH Terms] #8 Search Pediatrics [MeSH Terms] #9 Search child [MeSH Terms] Search (((school children [Title]) OR Child, Preschool [MeSH Terms]) OR Pediatrics [MeSH Terms]) OR child [MeSH Terms] #10 MeSH descriptor: [Dental Caries] explode all trees #11 MeSH descriptor: [Periodontal Diseases] explode all trees #12 MeSH descriptor: [Periodontitis] in all MeSH products #13MeSH descriptor: [DMF Index] explode all trees #14 MeSH descriptor: [Tooth Loss] explode all trees #15 (“edentulism”):ti,ab,kw #16 (Dental Status):ti,ab,kw #17 MeSH descriptor: [Oral Health] explode all trees #18 MeSH descriptor: [Oral Hygiene Index] explode all trees #18 #9 OR #10 OR #11 OR #12 OR #13 OR #14 OR #15 OR #16 OR #17 #19 #4 AND #8 AND #18

Initially two researchers (A.B. and B.A.) screened the titles and abstracts of the retrieved papers independently according to the inclusion and exclusion criteria. Any disagreements between the two reviewers were resolved by discussion with a third reviewer (A.M.B.). Potential eligible studies were then assessed in full for inclusion according to eligibility criteria. Additionally, manual searches on the reference list of the selected articles were carried out for identification of additional studies.

### Data extraction and quality assessment

The following information was recorded from the selected articles: first author, year of publication, sample size, country, study design, and quality assessment. The authors of the selected papers were contacted to provide further details when necessary.

Five researchers recorded the data independently using predefined excel sheets. First, duplicated titles and abstracts of the selected papers were initially omitted after revision. Second, manuscripts were selected based on titles and abstracts for further review using the full texts. The duplicate papers were identified using “find duplicate” option on endnote software and were deleted. The selection of full text articles was conducted by 5 authors according to the eligibility criteria.

Disagreements between the researchers were resolved by discussion. The Unweighted Kappa used to assess the consistency between the two researchers (B.A. and A.B.) during the selection of studies was 0.86.

### Quality assessment

Quality assessment was performed by the same two researchers using the Critical Appraisal Checklist for observational studies proposed by The Joanna Briggs Institute (JBI) to assess the internal validity and risk of bias of the studies [31]. The checklist for cohort, case-control studies and cross-sectional studies are made of 10, 10 and 8 items, respectively. Each item may score one point for the answer “yes” of each study and the score may vary from 0 to 10 (cohort and case-control studies) or 0 to 8 (cross-sectional studies). The papers were categorized as follows: low quality (0–3 scores), medium quality (4–6 scores); and high quality (7–10 scores).

### Data synthesis and statistical analysis

The meta-analysis was conducted to estimate pooled Odds Ratios (ORs) and 95% confidence intervals (95% CI) on the relationship of oral health conditions, demographic and socioeconomic characteristics with OHRQoL in children. Heterogeneity between studies was assessed using I^2^ statistics that is percentage of variation studies [32]. According to I^2^ index Random and fixed-effect methods were used for estimation pooled effect size in defined subgroups [33]. When I^2^ indexed is lower than 50%, the fixed effect model but if I^2^ index is higher than 50%, random effect model used for estimation pooled effect size [33].

The trend of pooled ORs according to country’s Human Development Index [34] (range = 0.761–0.939), sample size (range = 260–1134), year of study and age of participants (range = 3–12 years) was calculated by cumulative regression analysis. HDI data was obtained from World Bank data [35]. Potential publication bias was tested using the rank correlation of Begg’s test and Egger’s test [36, 37]. Publication bias among studies was statistically significant (*P* < 0.05). Therefore, Meta Trim test was performed to estimate pooled OR to eliminate the publication bias. The level of significance established for all analyses was 5% (*P* ≤ 0.05). Stata version 13.0 was used in all analyses (Stata Corporation, College Station, TX, USA).

## Results

### Study selection

Initially, 12,266 papers were identified through database searching and reference lists. Of them, 7428 studies were retained after removing duplicate references using “find duplicate” tool of endnote software. All titles and abstracts were reviewed, and 2586 were considered irrelevant and were excluded. The remaining 4842 articles were evaluated and further 1765 studies were excluded. The full text of the remaining 3077 articles was analysed for inclusion. Of them, 3066 studies were thereafter excluded according to the inclusion criteria. In the end, a total of 11 studies were included in this systematic review and meta-analysis [6, 38–47].

**Fig. 1:**
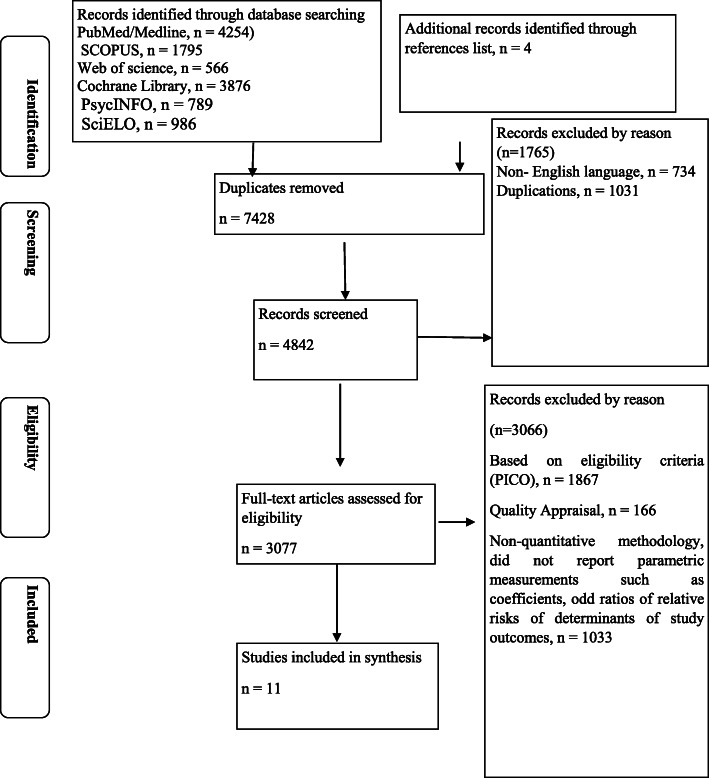
PRISMA flow diagram

Figure 1 demonstrates the review process.

### Study characteristics

The characteristics of the 11 selected studies are presented in Table 2. The sample size ranged from 103 to 1134 participants. Most studies were published between 2009 and 2013 and they predominantly conducted in Brazil. One cohort, one case-control and 9 cross-sectional studies were selected. Of the 11 studies, 6 were considered of high quality.

**Table 2:** Main characteristics of the studies selected

author	participants	Sample size	year	country	design	Quality of the evidence
[1]	Children	510	2009	*Hong Kong*	Case-control	Moderate
[2]	Children	286	2013	Brazil	Cross-section	High
[3]	Children	784	2013	Sri Lanka	Cross-section	Moderate
[4]	Children	103	2011	New Zealand	Cohort	High
[5]	Children	515	2012	Brazil	Cross-section	Moderate
[6]	Children	815	2019	Brazil	Cross-section	Moderate
[7]	Children	260	2011	Brazil	Cross-section	High
[8]	Children	638	2012	Brazil	Cross-section	Moderate
[9]	Children	456	2017	Brazil	Cross-section	High
[10]	Children	792	2010	Brazil	Cross-section	High
[11]	Children	1134	2014	Brazil	Cross-section	High

1. Du R, Yiu C, King N: **Health-and oral health-related quality of life among preschool children with autism spectrum disorders**. *European Archives of Paediatric Dentistry* 2019:1–9

2. de Paula JS, Leite ICG, de Almeida AB, Ambrosano GMB, Mialhe FL: **The impact of socioenvironmental characteristics on domains of oral health-related quality of life in Brazilian schoolchildren**. *BMC oral health* 2013, **13**(1):10

3. Nanayakkara V, Renzaho A, Oldenburg B, Ekanayake L: **Ethnic and socio-economic disparities in oral health outcomes and quality of life among Sri Lankan preschoolers: a cross-sectional study**. *International journal for equity in health* 2013, **12**(1):89

4. Shearer DM, Thomson WM, Broadbent JM, Poulton R: **Does maternal oral health predict child oral health-related quality of life in adulthood?** *Health and quality of life outcomes* 2011, **9**(1):50

5. Paula JS, Leite IC, Almeida AB, Ambrosano GM, Pereira AC, Mialhe FL: **The influence of oral health conditions, socioeconomic status and home environment factors on schoolchildren’s self-perception of quality of life**. *Health and quality of life outcomes* 2012, **10**(1):6

6. Gatto RCJ, Garbin AJÍ, Corrente JE, Garbin CAS: **The relationship between oral health-related quality of life, the need for orthodontic treatment and bullying, among Brazilian teenagers**. *Dental press journal of orthodontics* 2019, **24**(2):73–80

7. Abanto J, Carvalho TS, Mendes FM, Wanderley MT, Bönecker M, Raggio DP: **Impact of oral diseases and disorders on oral health-related quality of life of preschool children**. *Community dentistry and oral epidemiology* 2011, **39**(2):105–114

8. Martins-Júnior P, Vieira-Andrade R, Corrêa-Faria P, Oliveira-Ferreira F, Marques L, Ramos-Jorge M: **Impact of early childhood caries on the oral health-related quality of life of preschool children and their parents**. *Caries research* 2013, **47**(3):211–218

9. Chaffee BW, Rodrigues PH, Kramer PF, Vítolo MR, Feldens CA: **Oral health-related quality-of-life scores differ by socioeconomic status and caries experience**. *Community dentistry and oral epidemiology* 2017, **45**(3):216–224

10. Piovesan C, Antunes JLF, Guedes RS, Ardenghi TM: **Impact of socioeconomic and clinical factors on child oral health-related quality of life (COHRQoL)**. *Quality of Life Research* 2010, **19**(9):1359–1366

11. Tomazoni F, Zanatta FB, Tuchtenhagen S, da Rosa GN, Del Fabro JP, Ardenghi TM: **Association of gingivitis with child oral health–related quality of life**. *Journal of periodontology* 2014, **85**(11):1557–1565

### Meta-analysis

#### Oral health status and OHRQoL

##### Orthodontic treatment need and OHRQoL

**Fig. 2:**
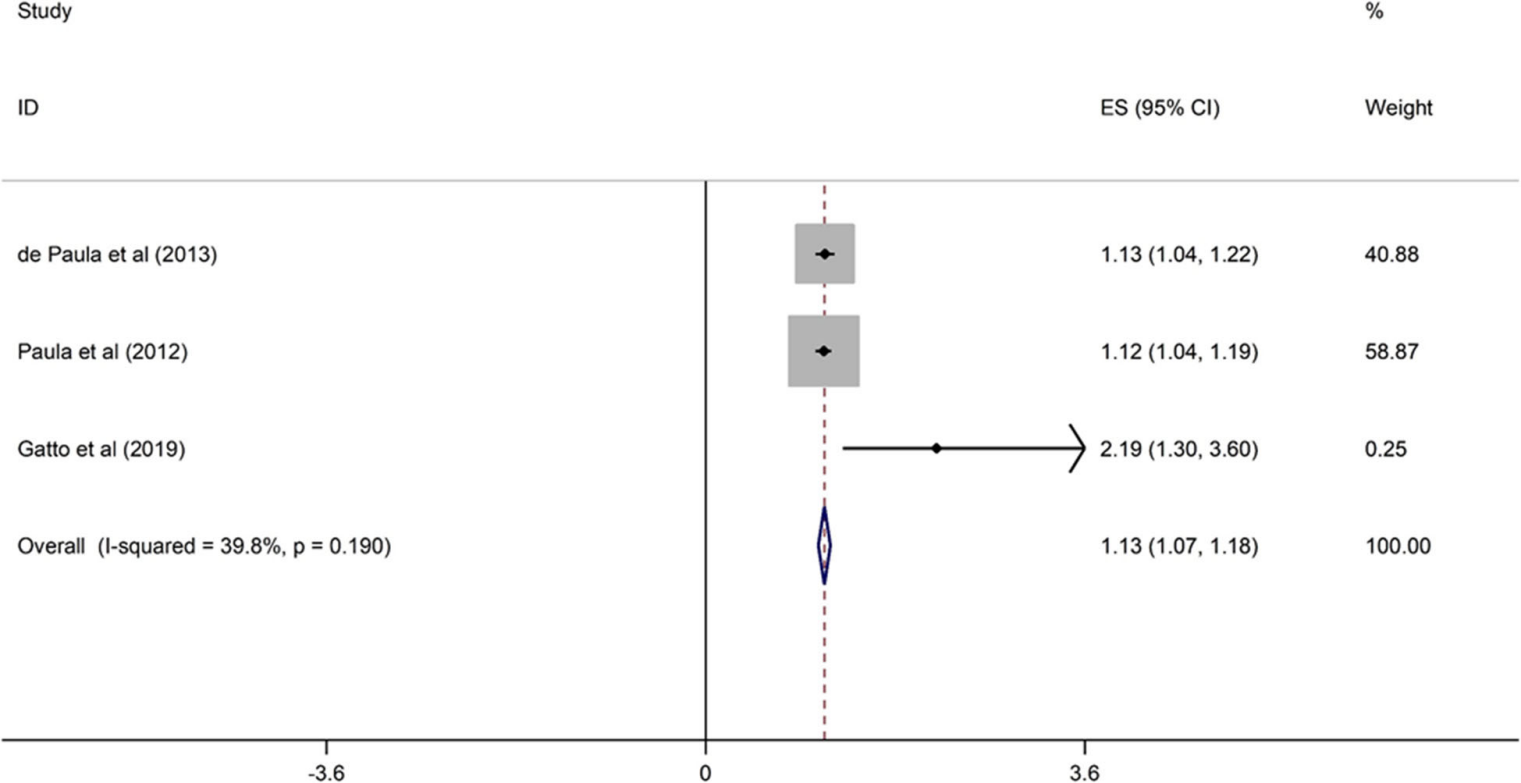
Forest plot of effect of orthodontic treatment need on OHRQoL among children

Three cross-sectional studies evaluated the relationship between orthodontic treatment need and OHRQoL among children [6, 39, 42]. All studies were conducted in upper middle-income countries between 2012 and 2019. The sample size ranged from 286 to 815 and two studies were of high quality. Children with orthodontic treatment need had 13% higher probability of poor OHRQoL than those without orthodontic treatment need (OR = 1.13, 95% CI = 1.07, 1.18) (Fig. 2).

##### Dental caries and OHRQoL

**Fig. 3:**
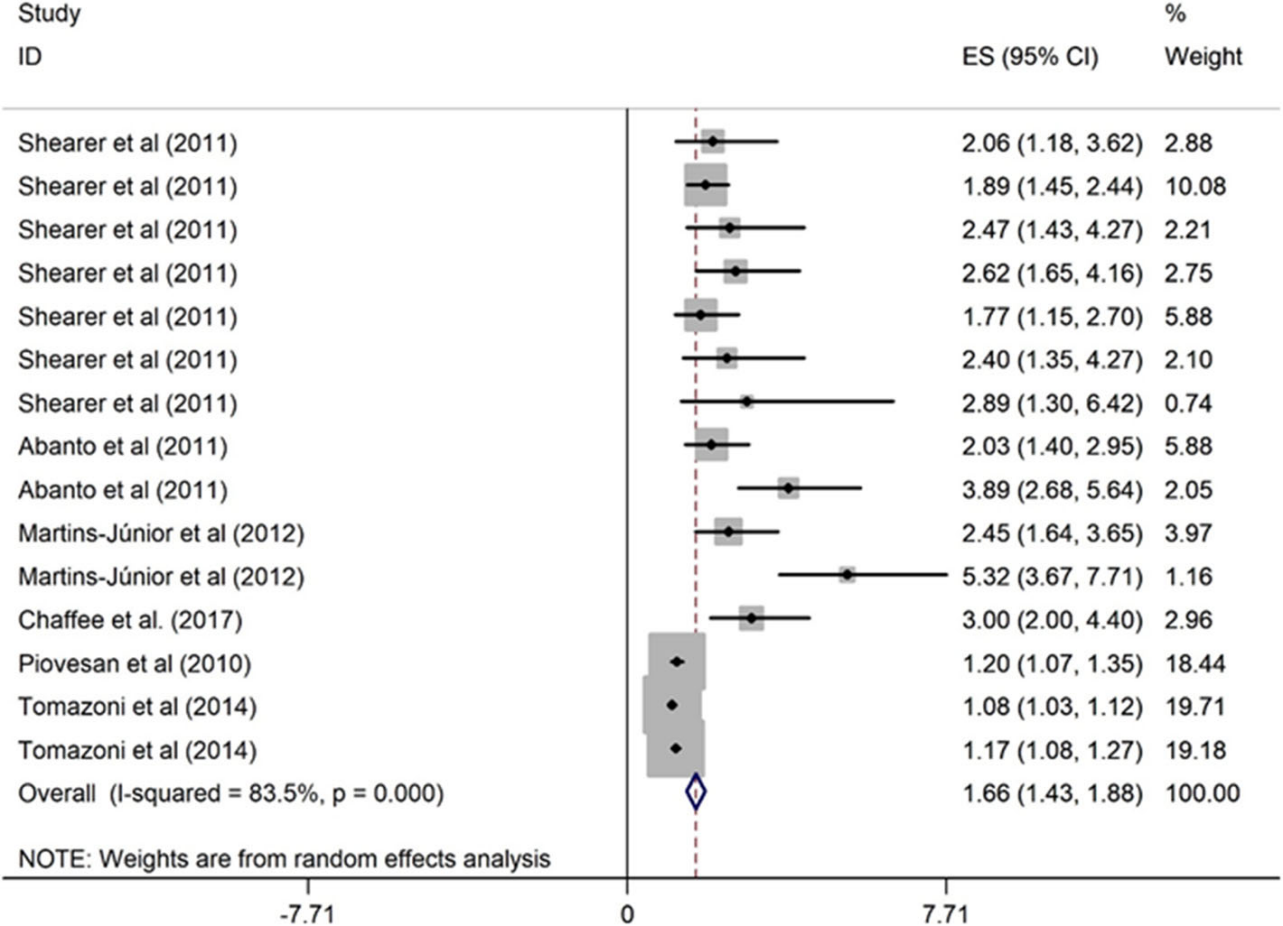
Forest plot of effect of dental caries on OHRQoL among children

The relationship between dental caries and OHRQoL in children was assessed in six studies [41, 43–47]. Of them, five studies were cross-sectional design [43–47], and one used cohort design [41]. Five studies were carried out in upper middle-income country [6, 39, 40, 45, 47] and one was conducted in higher-income country [41]. The studies were published from 2011 to 2017, and the sample size ranged from 260 to 1134. Three studies were of high quality and the other three were of moderate quality. Higher levels of dental caries was associated with poor OHRQoL. Children with more dental caries were 1.66 times more likely to have poor OHRQoL than caries-free children (OR = 1.66, 95% CI = 1.43, 1.88) (Fig. 3).

##### Periodontal disease and OHRQoL

**Fig. 4:**
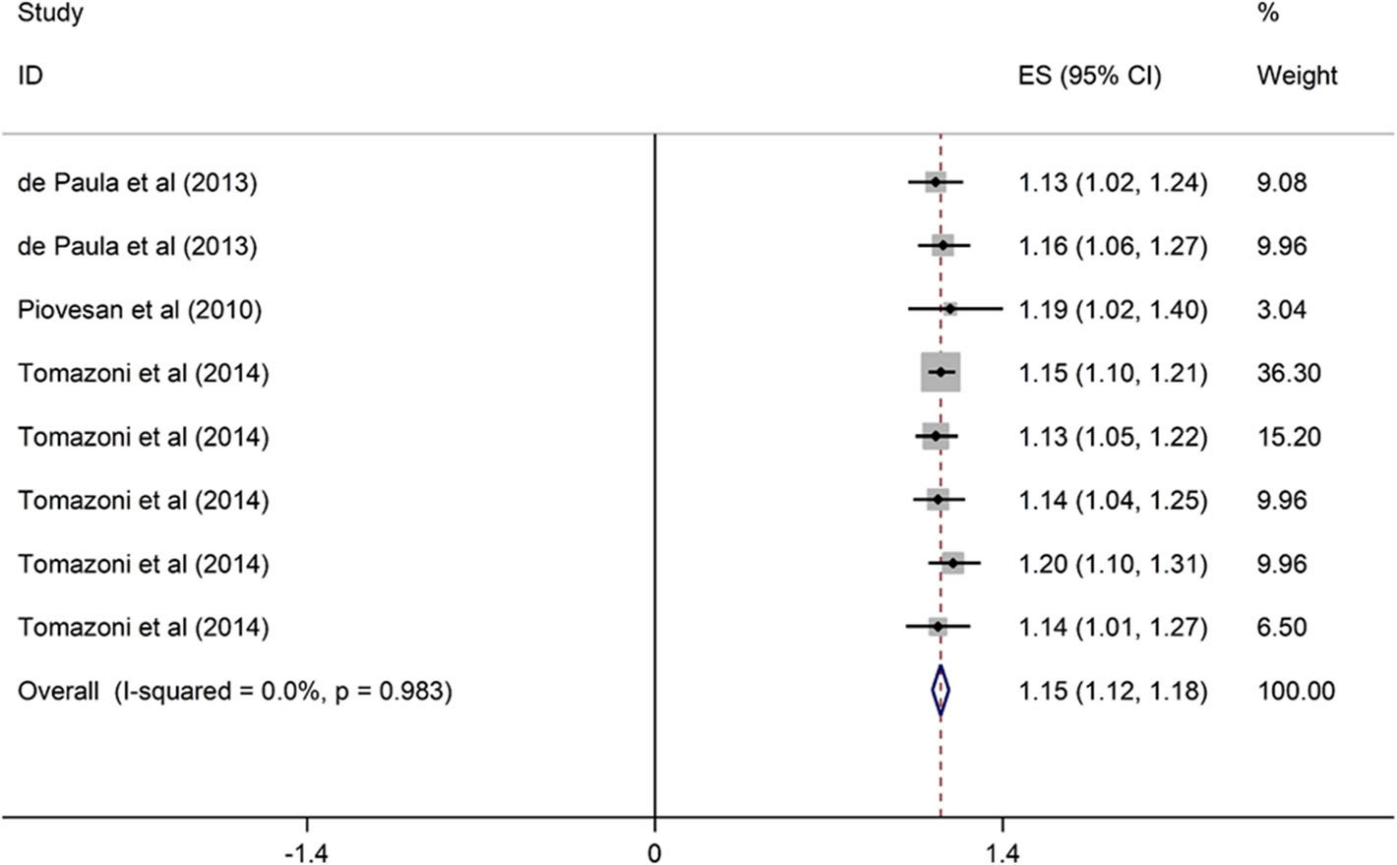
Forest plot of effect of periodontal disease on OHRQoL among children

Three cross-sectional studies conducted in upper middle-income countries assessed the association between periodontal disease and OHRQoL among children [39, 46, 47]. They were published from 2010 to 2014, and the sample size ranged from 286 to 1134. All three studies of high quality. Periodontal disease was significantly associated with poor OHRQoL in children (OR = 1.15, 95% CI = 1.12, 1.18) (Fig. 4).

#### Demographic and socioeconomic characteristics

##### Children’s age and OHRQoL among children

**Fig. 5:**
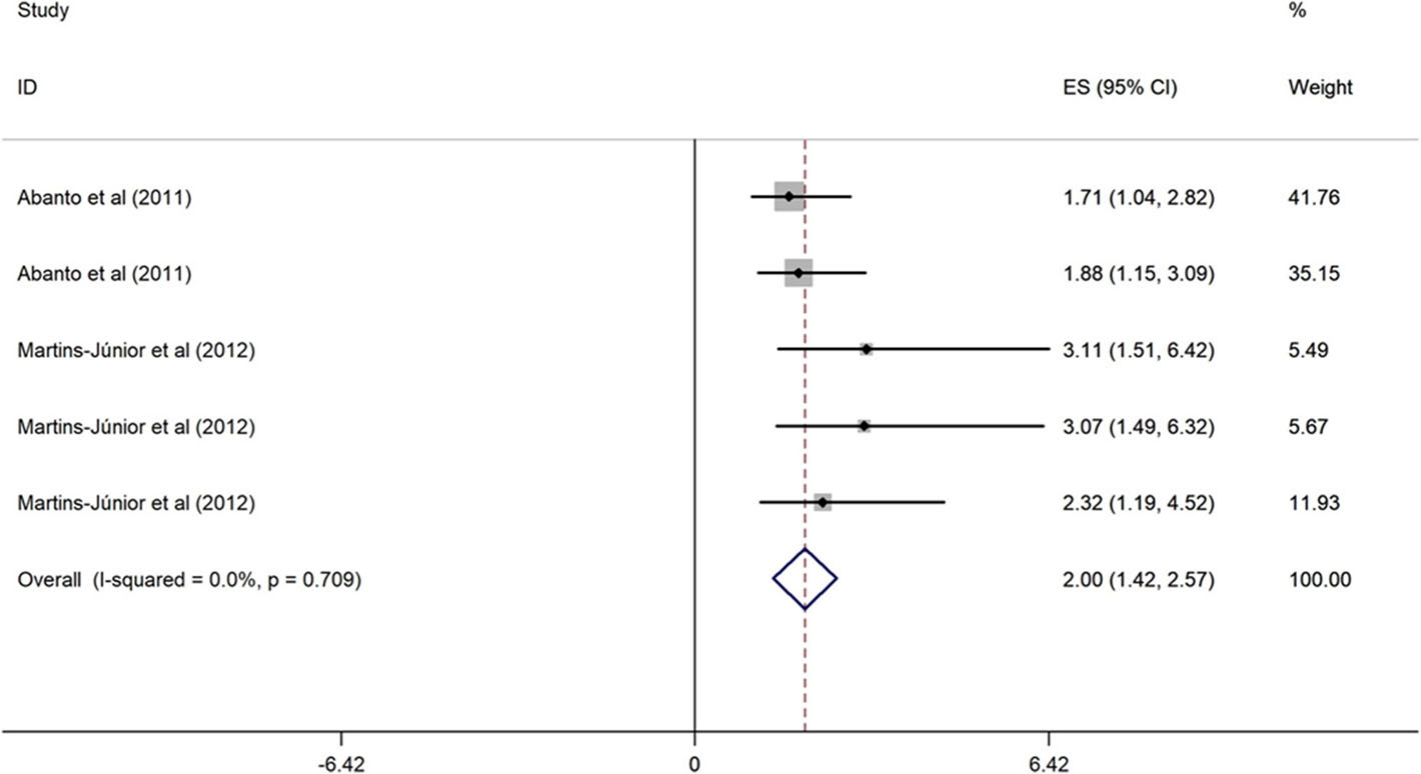
Forest plot of effect of children aged 3 on OHRQoL among children

**Fig. 6:**
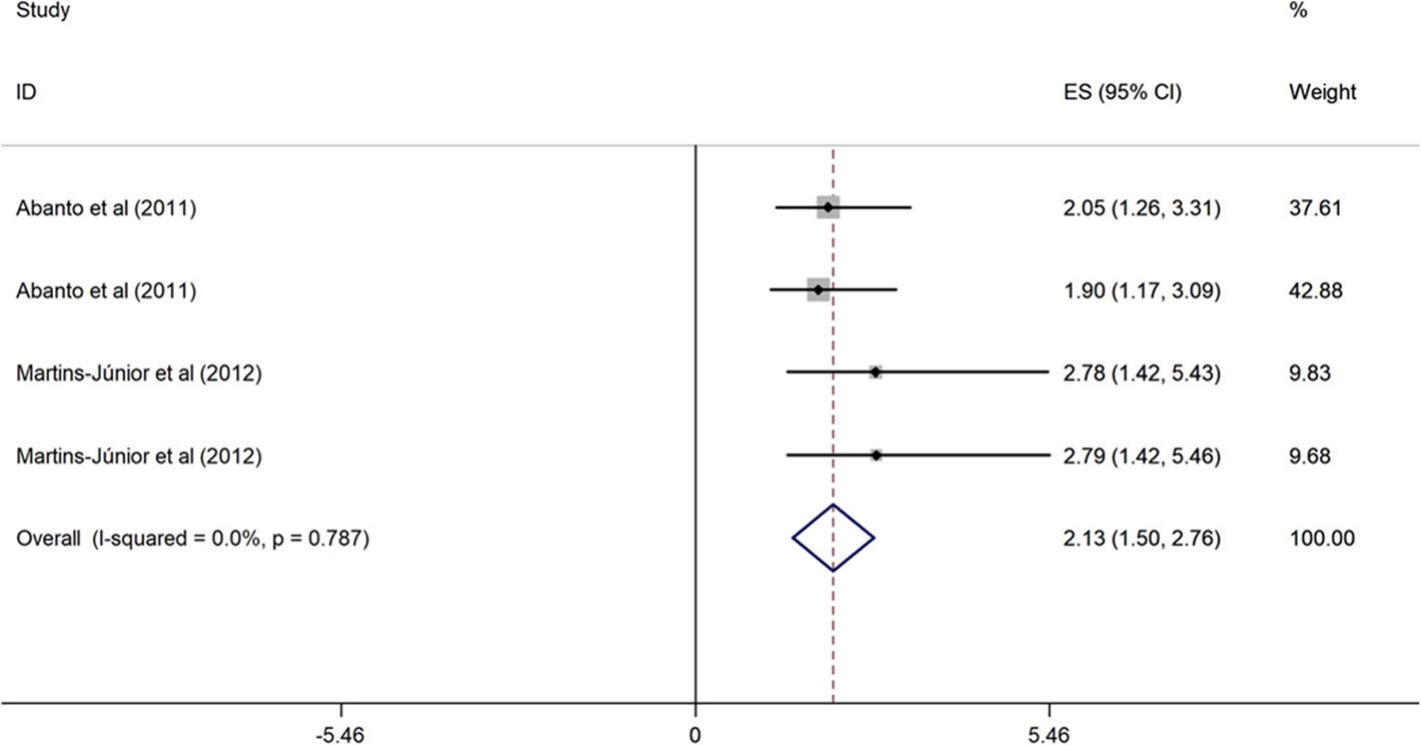
Forest plot of effect of children aged 4 on OHRQoL among children

**Fig. 7:**
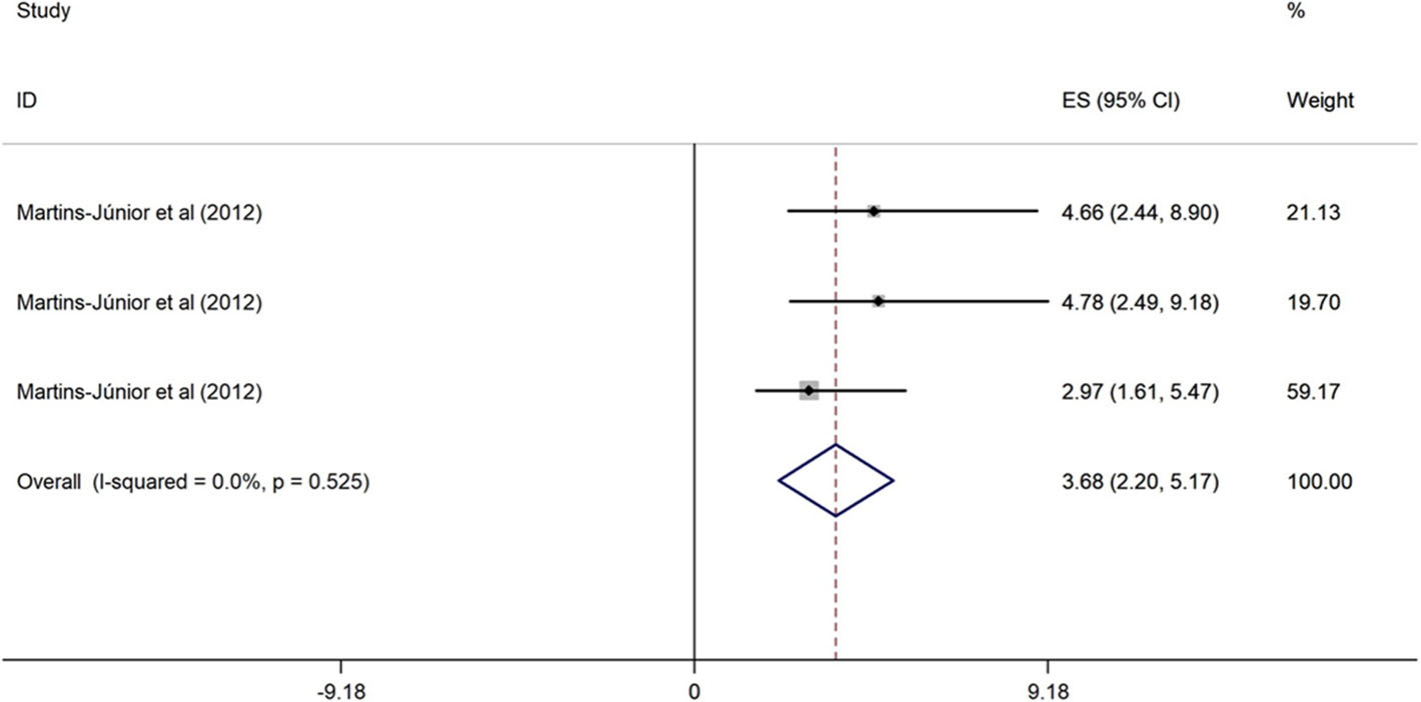
Forest plot of effect of hildren aged 5 on OHRQoL among children

Two cross-sectional studies conducted in upper middle-income evaluated the association between low children’s age (3–5 years vs. > 5 years) and OHRQoL [43, 44]. These studies were published from 2011 to 2012, and the sample size ranged from 260 to 638. The two studies were of high quality, and one research was categorized as poor quality. Children aged 3, 4 and 5 years were 2.00, 2.13 and 3.68 times more likely to have poor OHRQoL (OR = 2.00, 95% CI = 1.42, 2.57; OR = 2.13, 95% CI = 1.50, 2.76; and OR = 3.68, 95% CI = 2.20, 5.17) than children with 5 years-old (Figs. 5, 6 and 7).

##### Gender and OHRQoL

**Fig. 8:**
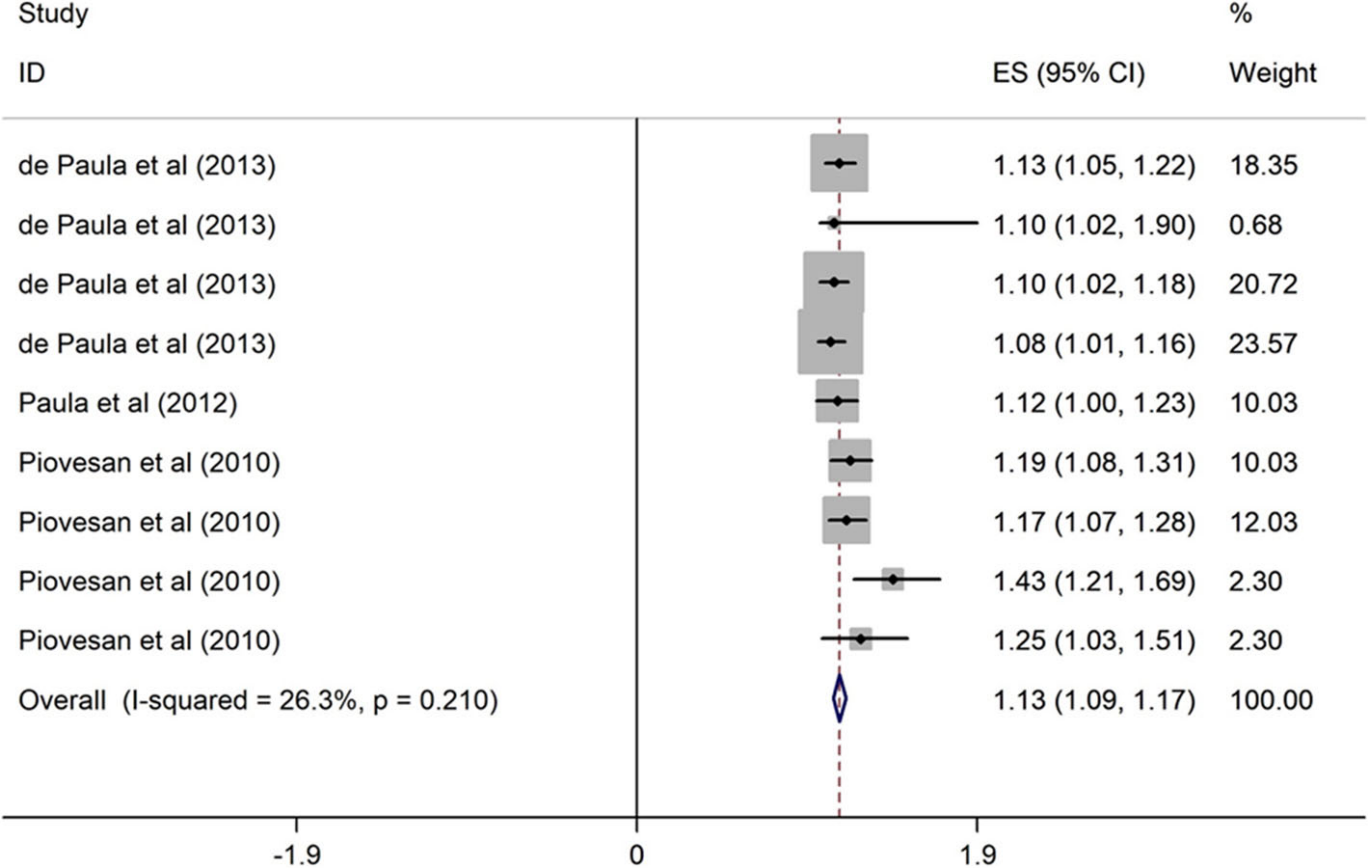
Forest plot of effect of gender on OHRQoL among children

Three cross-sectional studies conducted in upper middle-income countries [6, 39, 46] examined the association between gender and OHRQoL among children. The studies were published from 2010 to 2013, and the sample size ranged from 286 to 792. Two studies had high quality. There was a positive association between gender (girls vs. boys) and poor OHRQoL among children. Female children were 1.14 times more likely to have poor OHRQoL than male children (OR = 1.13, 95% CI = 1.09, 1.17) (Fig. 8).

##### Family income and OHRQoL

**Fig. 9:**
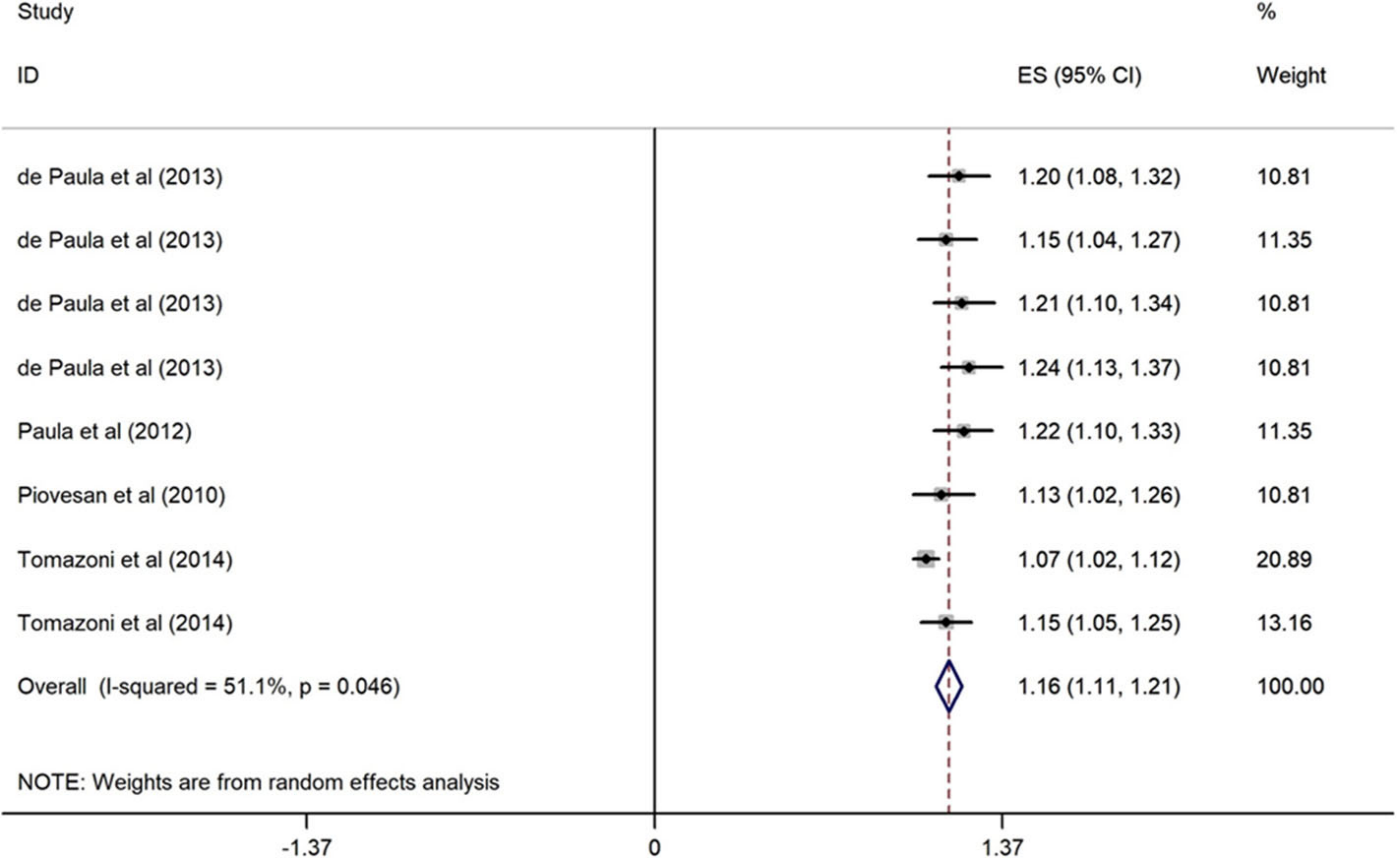
Forest plot of effect of family income on OHRQoL among children

Four cross-sectional studies carried out in upper middle-income countries (Brazil) evaluated the relationship between family income and OHRQoL among children. These studies were conducted between 2010 to 2014, and the sample sizes ranged from 286 to 1134 with a high-quality structured approach. All studies considered the threshold of $70 per month to classify the participants as low- (<$70) and high-family income (≥$70). Children from low-income families (< 70$) were 1.16 times more likely to have poor OHRQoL (OR = 1.16, 95% CI = 1.11, 1.21) (Fig. 9).

##### Maternal education and OHRQoL

**Fig. 10:**
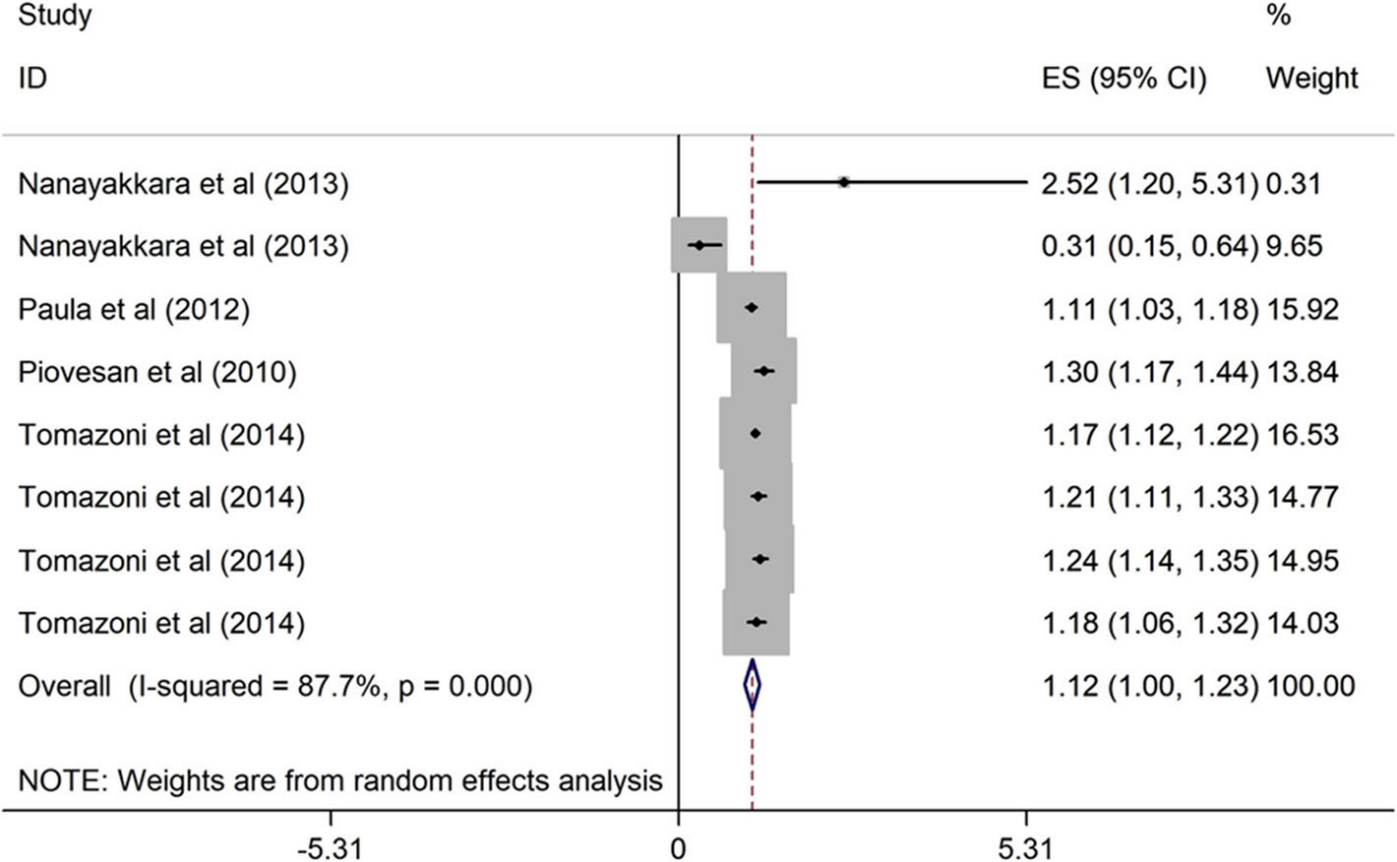
Forest plot of effect of maternal education on OHRQoL among children

Four cross-sectional studies [6, 40, 46, 47] carried out in upper middle-income countries evaluated the association between maternal education and OHRQoL among children. The studies were published from 2010 to 2014, and the sample size ranged from 515 to 1134. Three studies were considered of high quality. Lower maternal education (≤ 6 years of schooling) was significantly associated with poor OHRQoL (OR = 1.12, 95% CI = 1.00, 1.23) (Fig. 10).

##### Assessment of heterogeneity

**Fig. 11:**
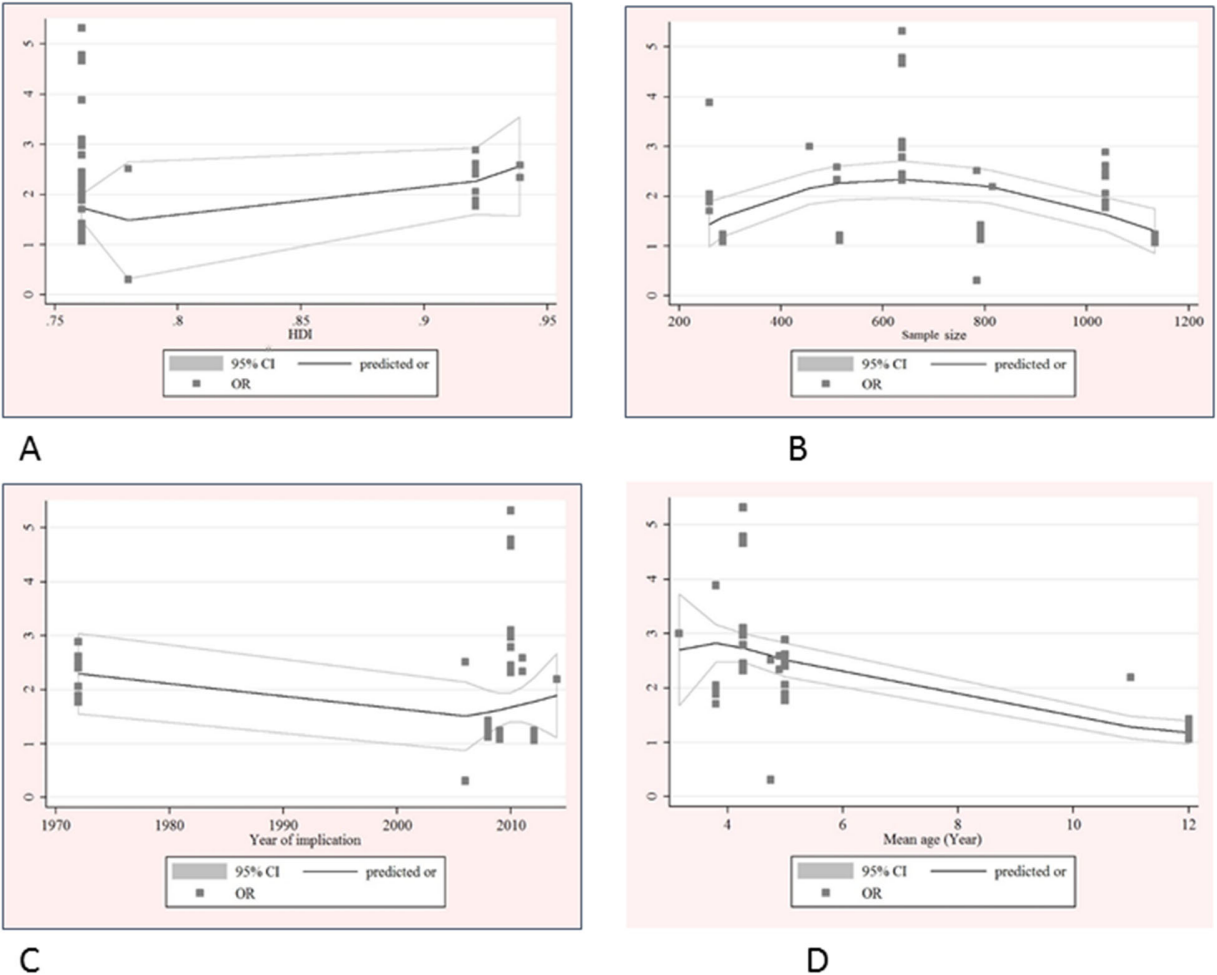
Meta-regression analysis for determine association between poor quality of life with HDI (**a**), sample size (**b**) and year of implication (**c**), children age (**d**)

Heterogeneity between studies was observed on the meta-analysis of dental caries (I2 = 83.5%, *P* < 0.001), family income (I2 = 51.1%, *P* = 0.046) and maternal education (I2 = 89.2%, *P* < 0.001) with OHRQoL. The meta regression analysis showed that country’s HDI (C = 1.45, *p* value = 0.03), study’s sample size (C = 2.32, *p* value < 0.001), year of study (C = 1.48, *p* value < 0.001) and age of children (C = 1.57, *p* value < 0.001) were significant characteristics that influenced the observed heterogeneities (Fig. 11).

##### Publication bias

**Fig. 12:**
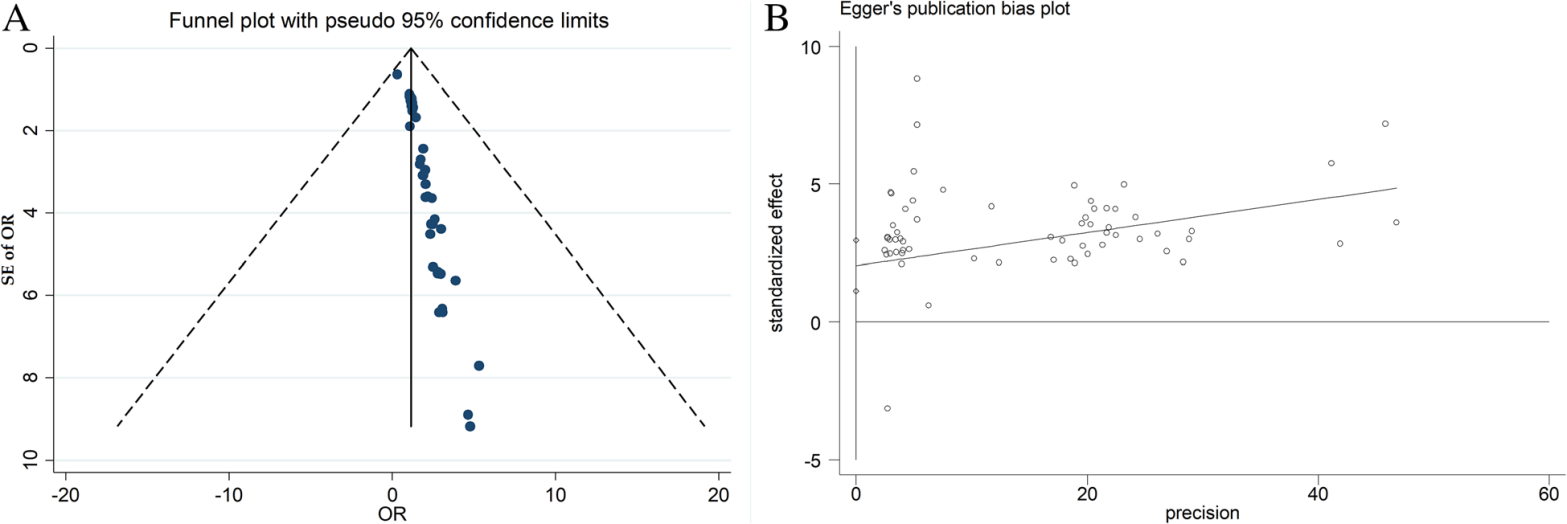
Determine publication bias by Beggs (**a**) and Egger’s (**b**) tests

**Fig. 13:**
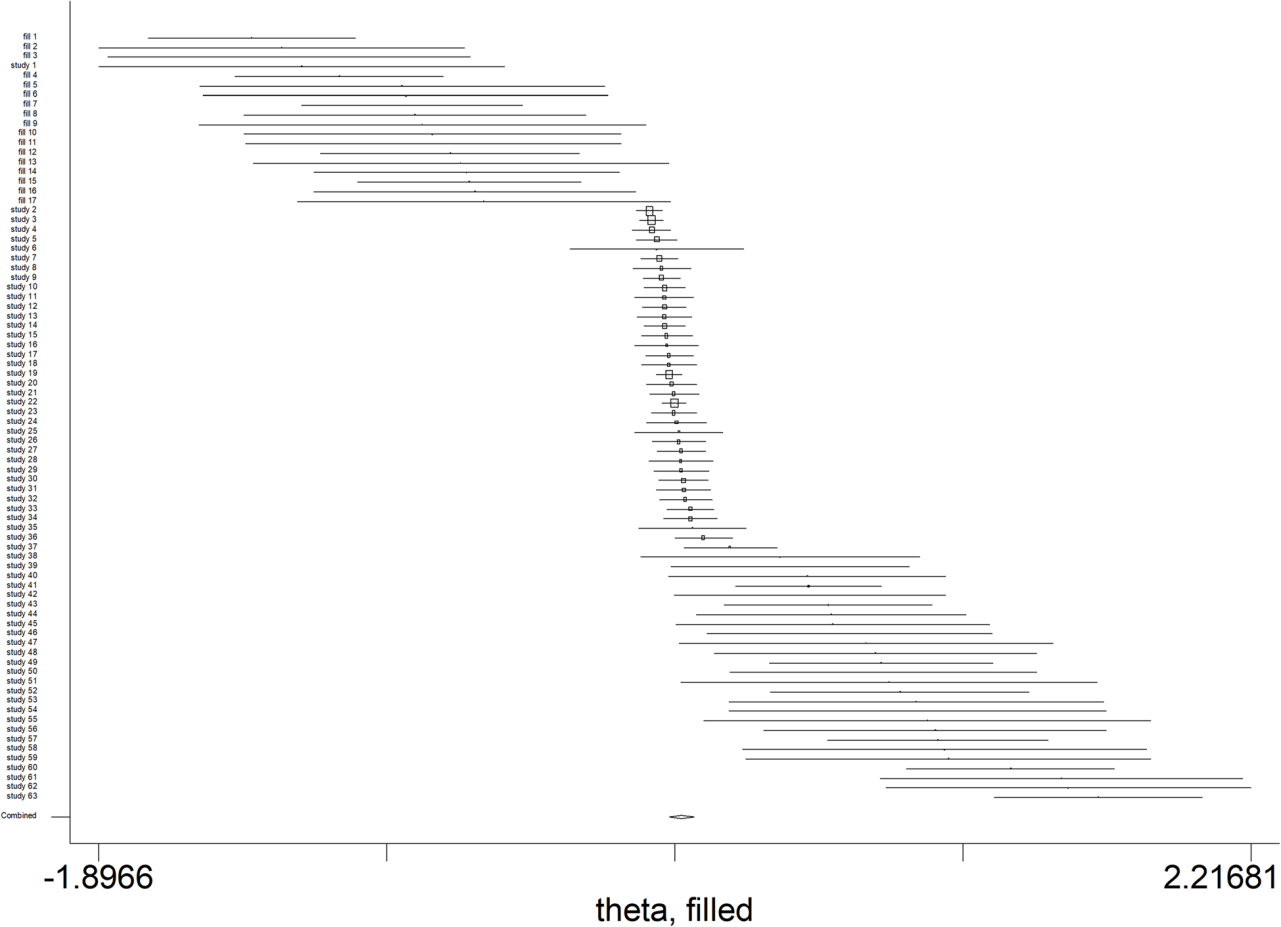
Meta trim plot for determine pooled OR without publication bias

Begg’s test and Egger’s tests showed evidence of publication bias (Begg’s test: *P* < 0.001; Egger’s test: *P* < 0.001) (Fig. 12). According to publication bias test, a significant publication bias among studies was noted (C = 2.38; *P*-value = 0.001) (Fig. 12). Therefore, metatrim analysis was performed in order to remove the effect of publication bias on the pooled OR. The meta-trim analysis revolved that the pooled OR 0.17 (95%CI, 0.13–0.21) in the random effect model (Fig. 13).

## Discussion

This study aimed to examine the possible relationship of oral health conditions, demographics and socioeconomic characteristics with OHRQoL in children.

Our findings suggest that poor oral health status, greater age, female gender and worse socioeconomic status were significantly associated with poor OHRQoL. These results are in accordance with previous research [43, 48, 49] that showed a correlation between socio-economic status, clinical status and OHRQoL in preschool children [25, 43]. In this study, children’s poor OHRQoL was strongly correlated with lower levels of maternal education (≤ grade 6). Piovesan et al. suggested that low household income was a risk factor for poor OHRQoL in children [46]. Besides, lower paternal and maternal educational level were associated with poor OHQoL of their children [46]. Female gender was also associated with poor OHRQoL, which is in agreement with previous studies [39, 46]. The possible explanation for this finding is the poor self-esteem and poor self-perception of oral health and body image among girls compared with boys [50–52]. According to Honkala et al. [53], female gender is significantly correlated with frequency of tooth brushing, and self-esteem among schoolchildren was also associated with a high socioeconomic status of the family. Foster Page et al. [52] reported that gender’s psychosocial characteristics can affect OHRQoL.

Moreover, a negative association between orthodontic treatment need and function limitation dimension of CPQ11–14 was reported. Functional limitation is composed of three items that assesses difficulty in eating and eating hot and cold foods; taken longer to eat a meal; difficulty in biting or chewing firm foods (e.g. apples, corn on the cob, steak); difficulty in opening the mouth wide; problems with pronouncing some words; difficulty with eating favorite foods; difficulty with drinking through a straw [50, 51]. Another research revealed that orthodontic treatment need was associated with poor OHRQoL and dental aesthetics [50].

Bleeding was also significantly related to emotional and social well-being domains of the CPQ11–14. These findings are consistent with those reported by Lopez and Baelum [54] on the association between periodontal disease and poor OHRQoL. Nevertheless, a different OHRQoL instrument (Oral Health Impact Profile OHIP) was used to assess the impact of periodontal diseases on OHRQoL.

Previous studies suggested the poor correlation between dental caries and oral symptoms and functional limitations domains of CPQ [6, 55]. Furthermore, orthodontic treatment need was the only clinical variable associated with OHRQoL [46, 56]. As a result, the orthodontic treatment need was mediated by the individual and socio environmental characteristics.

The reported data highlight the possible significant impact of social interventions and health promotion strategies. Such interventions could be beneficial to support the development of supportive environments for this population. They can also improve their health-related skills to enhance their health status and to decrease health inequalities [4, 25, 46]. Oral symptoms and functional limitations had the most significant impact on oral health conditions in terms of adolescents’ social relationships. (Agou et al.) [51]. According to Marmot and Bell, tackling the social determinants of health, including improving individual living status and structural drivers (e.g. laws, policies, economic conditions and cultural norms that shape and influence patterns of behaviour and individual capacities) are paramount. Furthermore, socio-environmental characteristics significantly affected children’s daily living status [57, 58]. As a result, promoting health status should be conducted for planning health promotion interventions in all social environments in which children live their lives, in order to promote supportive environments for them [59]. Policymakers of the health sector should address oral health at population level by reducing social inequalities.

### Limitations

This study has limitations that should be acknowledged. First, the included studies assessed OHRQoL using different questionnaires. Second, OHRQoL instruments might be considered generic questionnaires to assess the relationship between dental clinical measures and children’s OHRQoL, including periodontal disease. Third, articles published in non-English languages were excluded, which may have influence our results. Fourth, the selected articles investigated different predictors of OHRQoL and have used distinct analytical approaches, including univariate and multivariable regression methods. In addition, the categories of the predictors varied significantly between the selected papers, such as those employed to assess maternal education and family income. Therefore, specific inclusion criteria were necessary to include studies with similar methodological approaches in order to perform the meta-analysis. Fifth, some of the papers initially selected were thereafter excluded due to lack of information, as the corresponding author of those articles did not reply our contact to provide the information needed, such as the measures of association (e.g. odds ratio). Finally, the included studies did not inform the validity of the OHRQoL instruments.

## Conclusion

In conclusion, socioeconomic conditions, demographic characteristics and dental clinical measures might differently affect oral health-related quality of life among children. Our findings suggest that children aged 3–5 years, female children, those whose families earned less than 70 dollars per month, whose mothers had low education were more likely to poor OHRQoL. In addition, orthodontic treatment need, dental caries and periodontal disease were significantly associated with poor OHRQoL in children.

Considering the need to design and develop oral health strategies to improve children’s oral health, social and environmental conditions where the children live should be considered in planning, implementation and evaluation of oral health promotion activities. Also, further longitudinal studies should be conducted to determine causal relationships between the investigated predictors and OHRQoL.

## Data Availability

The datasets used and/or analyzed during the current study are available from the corresponding author on reasonable request.

## Abbreviations

QoL: Quality of life
DMFT: Decayed, missing and filled teeth
OHRQoL: Oral health-related quality of life
PRISMA: Preferred for systematic reviews and meta-analyses
PPD: Pocket probing depth
CAL: Clinical attachment level
JBI: The Joanna Briggs institute
OR: Odds ratio
CI: Confidence interval
PSI: Periodontal screening index
BOP: Bleeding on probing
TD: Tooth decay
ECOHIS: Early childhood oral health impact scale
MT: Missing teeth
